# Should the Use of Antitoxin in Tuberculosis Be Condemned from a Purely Scientific Point of View?

**Published:** 1900-08

**Authors:** Carl Von Ruck

**Affiliations:** Asheville, N. C.


### Should the Use of Antitoxin in Tuberculosis Be Condemned From a Purely Scientific Point of View?

BY DR. CARL VON RUCK, ASHEVILLE, N. C.

To attempt to answer the above question from a scientific basis only would require a review of all the pertinent data in bacteriology, experimental therapeutics and clinical medicine which have accumulated within the last twenty years; and having done this, we would find that our exact knowledge bearing upon the subject of toxins and antitoxins in general and of their relation to tuberculosis in particular, is insufficient to come to a definite conclusion.

As my available time does not permit me to attempt such a review, and finding the scientific facts too meager, I desire to open this discussion from the standpoint of the therapeutist rather than that of the scientist, referring to such facts as speak for or against an affirmative answer.

Presuming that my hearers are familiar with the subject of bacterio-therapeutics, as evolved and applied in the several infectious diseases, I may give my definition of an antitoxin, "as a specific remedy having the power to antagonize, antidote, neutralize or to destroy the harmful effect of a specific bacterial toxin," to which I may add, that as commonly applied and understood the term "antitoxin" refers to blood serum obtained from animals which have undergone a course of treatment or immunization with toxins derived from specific bacteria.

The principle of bacterio-therapeutics rests upon the established relation of certain pathogenic bacteria to certain infectious diseases, and upon observation, that with recovery from some of the infectious diseases the individual is protected for a longer or shorter period of time against reinfection, or recurrence of the disease.

This change from susceptibility to immunity is believed to have been brought about through the action of specific toxins derived from the specific germs during the course of the disease, and but for such acquired immunity, recovery from infectious diseases would seem impossible.

Experimental medicine has shown that in several of the infectious diseases a like change from susceptibility to immunity can be induced by the artificial introduction of specific bacterial toxins, and also that the blood serum and other tissues of the immunized animals have the power to antagonize or overcome the deleterious effects of the bacterial toxin, and thus was evolved the theory of toxins and antitoxins.

The hope that such immunization and production of antitoxins was a general law applying to all infectious diseases, has, however, not yet been idealized.

The production of antitoxic serums for tuberculosis has been attempted by several experiments and also by the writer; and a number of preparations have been put upon the market within the last five or six years. The chief credit for the labor in this direction is due to Professor Maragliano; but all the scientific data which he and others could show is the fact that the antitoxin serum for tuberculosis contains antitoxic properties of only a very feeble power, the degree attained being so, that a minimum fatal dose of crude standardized tuberculin can be neutralized by injection of an equal quantity of serum.

Professor Maragliano claims for his prepartion that its clinical application in tuberculosis stimulates the production of *new* antitoxins in the patient, and that it has therefore immunizing properties also, which I, however, have not been able to confirm, either with Maragliano's or my own preparation, nor with several others used in my animal experiments.

If we may compare the serum preparations for diphtheria with those now obtainable for tuberculosis, we find that for a successful result in diphtheria a serum of an antitoxic power of several thousand units is required; while the best that has been accomplished for tuberculosis is a power of only one unit, and if any of the available preparations have clinical value, the latter is not likely to be due to their antitoxic properties, and from other considerations presently to be refered to, it is an open question whether or not a true antitoxin for tuberculosis is desirable, as is the case with diphtheria.

The latter is an acute infectious disease, and in uncomplicated cases, recovery occurs frequently after a short, definite course of illness, when the patient becomes temporarily immune. The diphtheria bacillus is a most rapid grower, and undergoes rapid disintegration. The pathological alterations in diphtheria are confined to the mucous surfaces, and repair is rapid and complete, after convalescence. The acute symptoms bespeak an acute intoxication, and when, in uncomplicated cases, death ensues, it is the direct consequence of bacterial poisoning. If under such conditions we can intervene with a remedy of the nature of an antitoxin, we may thereby avert death.

In tuberculosis, on the other hand, we have, as a rule, a chronic infectious disease in which immunity does not occur; patients acquire no well marked toleration to toxins; on the contrary they are, and remain, highly sensitive to them, so that toxins may be applied with almost unerring certainty for diagnostic purposes in most minute doses.

The tubercle bacillus is an extremely slow grower, and resists solution or disintegration to an astonishing degree. Tubercle bacilli can be demonstrated in old, fibroid lesions, and even in encapsuled caseous foci many years after the tissues were first involved in tubercular disease, and long after the conditions for the life and growth of the bacteria have apparently disappeared. No solution of the tubercle bacillus has been possible, and the extraction of toxins from their bodies was only accomplished by the removal of their great quantity of fat, and even then the bacilli retain their form.

The pathological alterations in tuberculosis are usually deep-seated and interstitial; the tubercles themselves undergo either rapid caseation, or gradual fibroid transformation, and in either case leave a permanent effect.

The clinical symptoms do not suggest the poisoning of the patient with specific toxins. Tubercle formation may be in progress and large areas of tissue may contain them without general symptoms of intoxication being present and the patient's life is never threatened by the action of specific toxins.

With such radical difference in the specific germs in the pathology and in the natural course of the disease, to which others may be added, the question arises: Are there specific toxins liberated in the course of tuberculosis to an appreciable amount? And since toxins appear necessary for the production of both the natural and artificially induced immunity in all infectious diseases, may the deficiency of toxin formation in tuberculosis not account for the continued susceptibility to tubercular invasion of new tissues, and to the tuberculin test? My own studies and observations incline me strongly to take this view, which my experimental results and therapeutical observations confirm.

I am well aware that the general symptoms in the course of pulmonary tuberculosis in all its stages have been attributed, and are still being attributed, to specific bacterial toxins, whether of the tubercle bacillus itself or of other associated germs; but I consider it a vital objection to the explanation that toxins from the tubercle bacillus are responsible for the general symptoms, when it can be shown that months after such symptoms have been present, the tubercular subject still shows general and local reactions to most minute doses of the specific toxins; while with the gradual introduction of the latter we can often reach 100 times the amount of the ordinary test dose in so short a time as a month, and do this without producing the slightest symptom of any kind. Surely, if the tubercular patient had been absorbing like toxins from his tubercular lesions for months or even years, he would thereafter not show reaction to a small fraction of a milligram of toxins.

This is not the time and place to give you my views as to the cause of the general symptoms, and especially the fever which we observe in the various stages of pulmonary tuberculosis, and which I have recorded in an article on fever in the January number of the *Journal of Tuberculosis,* on page 94.

To conclude my remarks, I may say again, that inasmuch as toxins are necessary to produce immunity, and since a true antitoxin will antagonize, neutralize or destroy the toxins, the latter could only have a place in the therapy of infectious diseases having an acute course during which the life of the patient is in jeopardy by excessive production of toxins, which is not the case in tuberculosis, and certainly not in its chronic form.

In order to justify the use of a pure antitoxin in tuberculosis, it must first be shown that in tuberculosis specific toxins from tubercle bacilli are present which have an unfavorable effect on the course of the disease.

If, however, it can be shown that the serum preparations, which we call antitoxins, although having a limited antitoxic property, have also the power of inducing a change from susceptibility to immunity, and act therefore in the same manner as do toxins, then of course the use of such serum cannot be condemned either from a scientific or therapeutic standpoint.

So far it has not been shown that any of the antitoxic serums, which have been produced by Maragliano as well as by others and by myself, have such properties to a degree that they produce even partial or slight degrees of immunity; and inasmuch as such results have been accomplished by the direct injection of toxins, and their administration appears to be perfectly safe and is attended by excellent clinical results, the direct immunization with toxins is justified from a scientific as well as from a therapeutic point of view.

